# Economic Evaluations of Kidney Paired Donation, ABO-Incompatible Kidney Transplantation, and Maintenance Dialysis in Different Resource Settings: A Systematic Review

**DOI:** 10.7759/cureus.114153

**Published:** 2026-08-08

**Authors:** Rahul Yadav, Swati S Jha, Khushboo Saxena, Manish R Balwani, Pranjal Kashiv, Maulik A Patel, Mohan Patel, Subho Banerjee, Divyesh Engineer, Jigar Shrimali, Nikunj Rout, Vivek B Kute

**Affiliations:** 1 Nephrology, Institute of Kidney Diseases and Research Center-Institute of Transplantation Sciences (IKDRC-ITS), Ahmedabad, IND; 2 Clinical Research, Case Western Reserve University, Ohio, USA; 3 Nephrology, Saraswati Kidney Care Center, Nagpur, IND; 4 Nephrology, Index Medical College, Hospital and Research Center, Indore, IND; 5 Nephrology, Shankus Hospital, Mehsana, IND; 6 Nephrology, Krishna Institute of Medical Sciences (KIMS) Manavata Hospital, Nashik, IND; 7 Nephrology, Renus Kidney Hospital, Ahmedabad, IND; 8 Nephrology, Kalinga Institute of Medical Sciences, Bhubaneswar, IND; 9 Treasurer, Indian Society of Organ Transplantation, Ahmedabad, IND

**Keywords:** abo incompatible kidney transplant, icer, kidney paired donation, kidney transplant, maintenance dialysis, quality-adjusted life years (qaly), transplant economics

## Abstract

Compared with long-term dialysis, kidney transplantation offers substantially better patient survival; however, donor scarcity and immunological barriers such as blood-group and human leukocyte antigen (HLA) incompatibility keep many candidates from ever reaching transplant. Kidney paired donation (KPD) works around this barrier by matching incompatible pairs into exchange chains, whereas ABO-incompatible (ABOi) transplantation removes the barrier directly through desensitization protocols. Little is known about how these two strategies compare economically, or whether any advantage they hold up when resource availability differs, since almost all published cost data originates from high-income settings. Our objective was to locate, critically evaluate, and pool the available economic evidence on KPD, ABOi living-donor transplantation, and maintenance dialysis in adult end-stage kidney disease (ESKD) patients, examining in particular whether conclusions differ among high-, middle-, and low-resource countries. This review followed the PRISMA 2020 framework, incorporating its equity-focused reporting extension. Literature was retrieved from three databases (PubMed/MEDLINE, Embase, and the Cochrane Library) covering economic evaluations (both full and partial) published from 2016 through 2025. Screening, data extraction, and methodological quality assessment were each carried out independently by a pair of reviewers, with appraisal tools selected to match study design: the Consensus on Health Economic Criteria (CHEC) for cost studies, the Philips checklist for decision-analytic models, and the Newcastle-Ottawa Scale (NOS) for observational cohorts. Each included study was then classified by the World Bank income tier and by the specific type of economic analysis it employed.

Ten eligible studies spanning six countries were identified: the United States, Netherlands, Sweden, Norway, United Arab Emirates, and Iran, with seven originating from high-income settings and the remaining three from Iran, the sole upper-middle-income country represented; no low- or lower-middle-income data were found. Across every study, the conclusion pointed the same way: transplantation proved more economical than dialysis. KPD carried a similar cost burden to other transplant modalities and produced savings of approximately US$100,000 per transplant relative to continued dialysis; in the Netherlands, a paired exchange program raised discounted quality-adjusted life years (QALYs) from 6.42 to 9.65, with a net gain of 4.05 QALYs once altruistic donor chains were factored in. ABOi transplantation, though costlier than ABO-compatible (ABOc) transplantation, remained cost-effective against dialysis at US$59,564 per QALY gained. Iranian data showed markedly favorable cost-effectiveness at US$1,744 per QALY. The lower absolute ratios seen in Iran are best attributed to reduced local healthcare costs rather than any fundamental difference in the underlying economic argument.

Across all three comparisons, KPD, ABOi transplantation, and dialysis, transplantation consistently emerged as the more cost-effective route to renal replacement therapy (RRT). KPD stood out in particular, generating cost savings, health gains, and sparing patients the added expense of desensitization. The most notable limitation of the current evidence base is its near-total absence from lower-resource countries, precisely the settings where questions of affordability carry the greatest weight.

## Introduction and background

Background

End-stage kidney disease (ESKD) is the terminal, irreversible stage of chronic kidney disease (CKD), which itself affects an estimated 697.5 million people worldwide, a global prevalence of roughly 9.1%, and accounts for 1.2 million deaths annually, with the burden concentrated in lower-resource settings [1]. Access to the renal replacement therapy (RRT) that sustains life in ESKD remains profoundly unequal: millions of patients who need it, particularly across low-income Asia and Africa, never receive it, and global RRT demand is projected to more than double by 2030 [2]. Clinical and economic decisions in ESKD care are therefore inseparable from the resource context in which they are made.

Among available RRT modalities, kidney transplantation is the treatment of choice for eligible patients, conferring a substantial long-term survival advantage over maintenance dialysis; a large systematic review has documented lower mortality, fewer cardiovascular events, and better health-related quality of life among transplant recipients than among dialysis-dependent patients [3,4]. Maintenance dialysis, particularly in-hospital hemodialysis, is the single costliest component of ESKD management, and redirecting eligible patients toward transplantation has long been recognized as a means of curbing this rising financial burden [5].

Realizing this potential is limited first by organ shortage, and in living-donor programs, further compounded by immunological incompatibility: roughly one-third of willing, medically suitable donors are excluded for being ABO-incompatible (ABOi) with their intended recipient, sensitized against them, or both [6]. Two strategies convert these otherwise unusable donor-recipient pairs into successful transplants, and diverge sharply in how they do so economically. Kidney paired donation (KPD), also termed kidney exchange, sidesteps the immunological barrier altogether by linking two or more incompatible pairs so that each recipient receives a compatible organ from a different pair's donor, achieving a compatible graft without the cost of antibody reduction. ABOi transplantation instead crosses the barrier directly through a structured desensitization protocol, combining plasmapheresis or immunoadsorption with anti-CD20 therapy, carrying the full resource cost of that protocol. Both strategies expand the effective living-donor pool, and even incompatible living-donor transplantation confers a clear survival advantage over indefinite wait-listing [7]. In high-volume living-donor programs, KPD has been characterized as both cost-effective and legally sound, expanding living-donor transplantation by an estimated 25-35% [8].

Economic evaluations in this field typically express findings using a small set of standard metrics. The incremental cost-effectiveness ratio (ICER) is the additional cost incurred per additional unit of health benefit gained, most often expressed as cost per quality-adjusted life-year (QALY), where a QALY represents one year of life adjusted for health-related quality of life. An intervention is generally considered "cost-effective" when its ICER falls below a prespecified willingness-to-pay threshold, the maximum amount a health system or decision-maker is prepared to pay for one additional unit of health gain, which varies substantially by country and income setting.

Rationale

Despite this operational progress, the economic evidence supporting KPD and ABOi transplantation is concentrated almost entirely in high-income health systems, where currencies, cost structures, willingness-to-pay thresholds, and dialysis-financing arrangements differ substantially from those in low- and middle-income countries (LMICs). This mismatch matters most where the stakes are highest: in lower-resource settings, where the RRT access deficit is widest, the risk of commercial exploitation in transplantation is most acute, and the marginal value of each additional transplant is greatest, large-scale economic data on KPD, ABOi transplantation, and maintenance dialysis remain scarce or absent [2,6,8]. National exchange programs have shown that these strategies are operationally feasible at scale, but whether they are economically justified across the full spectrum of resource contexts is a question no prior synthesis has addressed. Mapping where robust economic evidence exists, and where it does not, carries direct relevance for clinicians, payers, and policymakers weighing these strategies under resource constraints and helps define priorities for future research, particularly in LMIC settings where the burden is greatest and the evidence thinnest.

Objectives

This review was designed to answer a single overarching question: in adults with ESKD, what is the comparative economic profile of KPD, ABOi living-donor transplantation, and maintenance dialysis, and how does the available economic evidence differ across high-, middle-, and low-resource settings?

Specifically, our objectives were to systematically identify, appraise, and synthesize economic evaluations of KPD, ABOi kidney transplantation, and maintenance dialysis in adults with ESKD, and to compare their economic profiles across varying resource settings.

## Review

This review was conducted and reported in accordance with the Preferred Reporting Items for Systematic Reviews and Meta-Analyses (PRISMA) 2020 statement [9]. Because the review compares health-economic outcomes across heterogeneous resource settings, reporting was supplemented by the PRISMA-Equity (PRISMA-E) extension [10], which adds structured guidance for reporting equity-relevant variables, such as socioeconomic and geographic characteristics of study populations and settings, so that differences in economic outcomes across income groups can be assessed systematically rather than incidentally. This review was not registered with PROSPERO.

Eligibility criteria

Eligibility was specified using the population, intervention, comparison, outcome, and study (PICOS) framework adapted for economic evaluations (Table 1).

**Table 1:** PICOS framework PICOS: population, intervention, comparator, outcome, and study design; CKD: chronic kidney disease; KRT: kidney replacement therapy; LDKT: living donor kidney transplant; ICER: incremental cost-effective ratio; QALY: quality-adjusted life years; DALY: disability-adjusted life years

Parameter	Inclusion criterion
Population (P)	Adults with kidney failure (CKD G5) requiring kidney replacement therapy
Intervention (I)	Kidney paired donation (KPD); ABO-incompatible living-donor kidney transplantation (ABOi LDKT); or maintenance dialysis (hemodialysis/peritoneal dialysis)
Comparator (C)	Any alternative KRT modality (e.g., compatible LDKT, deceased-donor transplantation, dialysis, waiting list) or no comparator (cost-description studies)
Outcomes (O)	≥1 quantitative economic outcome: total or incremental cost, ICER, cost per QALY or DALY, benefit-cost ratio, net present/monetary value, or willingness to pay
Study design (S)	Full economic evaluations (cost-effectiveness, cost-utility, cost-benefit, and cost-minimization analyses) and partial or comparative cost analyses, conducted either as decision-analytic models (Markov, discrete-event simulation or decision-tree) or alongside empirical data (prospective or retrospective cohort, registry or cross-sectional studies, including stated-preference/contingent-valuation analyses). Studies reporting only clinical outcomes, narrative or systematic reviews, editorials, commentaries and conference abstracts were excluded
Setting	Any country; classified by World Bank income group [11] to permit cross-resource-setting comparison

Resource settings in this review were defined according to the World Bank's income classification system, which stratifies national economies into four categories based on gross national income (GNI) per capita using the Atlas method: low-income (GNI per capita ≤ US$1,145), lower-middle-income (US$1,146-4,515), upper-middle-income (US$4,516-14,005), and high-income (>US$14,005) [11]. Studies were included irrespective of country income level. Only studies published between 2016 and 2025 were included. The synthesis boundary for what constitutes an eligible “economic outcome” was prespecified as a broad quantitative economic-outcome definition (any of the outcomes listed above), rather than a strict cost-utility (ICER per QALY) definition.

Exclusion Criteria

The exclusion criteria included the following: narrative reviews, editorials, commentaries and protocols; conference abstracts; studies reporting clinical outcomes only with no economic component; duplicate datasets (most complete report retained); and non-English reports.

Information Sources and Search Strategy

Electronic searches were run in PubMed/MEDLINE from 2016 to 2025, supplemented by Embase and the Cochrane Library. Three thematic strands were built: (A) KPD economics, (B) ABOi transplantation economics, and (C) maintenance-dialysis economics. Each combined Medical Subject Headings (MeSH) with free-text title/abstract keyword clusters was joined by Boolean operators, then intersected with an economic-evaluation term block (cost, cost-effectiveness, cost-utility, economic evaluation, QALY, disability-adjusted life year (DALY)). The full, reproducible strategy for each database is provided in the Appendices.

In addition to the electronic database search, the reference lists of all studies assessed at full-text stage were manually screened (citation/snowball searching) to identify additional eligible studies not captured by the database search.

Data Collection Process and Data Items

Two reviewers independently extracted data in duplicate, using a piloted standardized form; discrepancies were resolved by discussion, with unresolved disagreements adjudicated by a third reviewer. Where a study reported more than one analysis, the base-case estimate was taken as primary, with remaining estimates retained as secondary data. Authors' own cost-effectiveness conclusions were recorded as reported, without reinterpretation. Where data were missing, graphically reported only, or ambiguous, corresponding authors were contacted.

For each study, a common set of descriptive, clinical and methodological variables was extracted to populate the evidence tables and to enable the cross-setting comparison central to this review. These fell into three groups.

Study identifiers: first author, year, country, World Bank income group

Clinical and design descriptors: design, sample size, study period, follow-up, index intervention and comparator

The economic parameters: proper-analytic perspective (payer, provider, patient, or societal), model type (Markov, discrete-event simulation, decision tree, or trial-/cohort-based), time horizon, discount rates for costs and outcomes, reporting currency and price year, the willingness-to-pay or cost-effectiveness threshold applied, and the cost components captured. Any assumptions about missing or unclear parameters were stated explicitly.

Methodological Quality and Critical Appraisal

Given the heterogeneity of study designs included in this review, methodological quality was assessed using design-specific critical appraisal instruments: the Consensus on Health Economic Criteria (CHEC) list [12] was applied to trial and cohort based economic evaluations, the Philips checklist [13] was applied to model-based economic evaluations, and the Newcastle-Ottawa Scale (NOS) [14] was applied to observational cohort studies without a formal economic evaluation component. Each instrument is independently validated for its respective study design; this tool-matching approach is consistent with recommended practice for systematic reviews incorporating heterogeneous economic evaluation designs.

Critical appraisal used design-matched instruments (CHEC, Philips checklist, NOS) and scored per original item-level grades (Y/P/N/U; see supplementary file in the Appendices for the full scoring legend and item-to-domain mapping). Domain-level percentage thresholds (≥75% low risk, 55-74% some concerns, <55% high risk) were set a priori by reviewer consensus.

Data Synthesis

A structured narrative synthesis was undertaken, following the Synthesis Without Meta-analysis (SWiM) reporting guideline [15]. This synthesis approach and its reporting follow the nine reporting items recommended by the SWiM guideline [15], including grouping rationale, standardized presentation of effect direction, methods for combining results within groups, and handling of studies reporting multiple outcomes, as described above. Findings were grouped by intervention domain (KPD, ABOi transplantation, maintenance dialysis) and stratified by World Bank income classification. Within each stratum, synthesis proceeded by direction of the cost-effectiveness conclusion, an effect-direction and vote-counting approach. 

Certainty of the Evidence

The strength of each domain-level conclusion was characterized qualitatively from the consistency of cost-effectiveness findings across studies, the results of the critical appraisal, and the applicability of each study to its income setting.

Results

Study Selection

**Figure 1:**
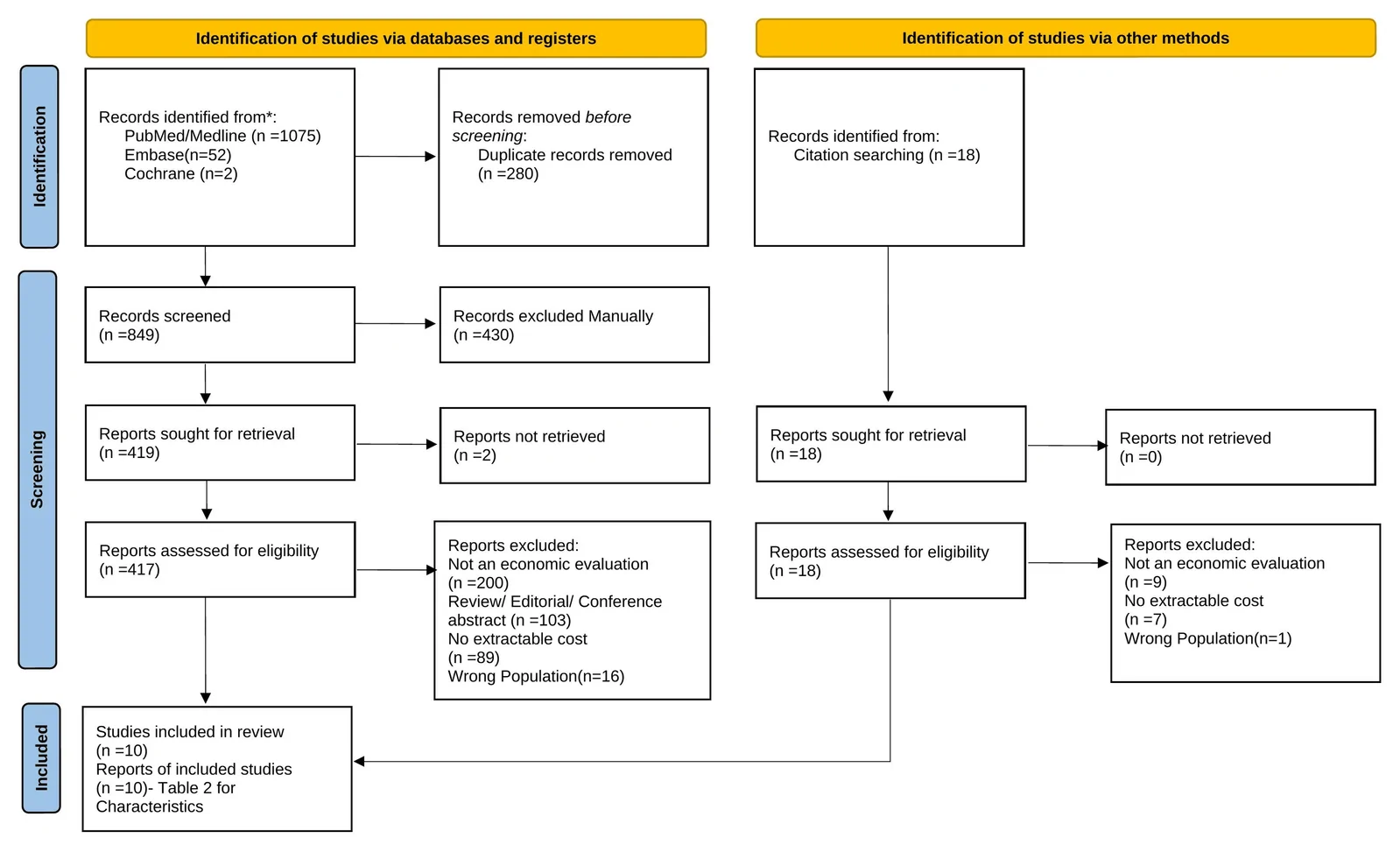
PRISMA flow diagram PRISMA: Preferred Reporting Items for Systematic Reviews and Meta-analyses

Searches of the electronic databases yielded 1,129 records; 1,075 from PubMed/MEDLINE, 52 from Embase, and two from the Cochrane Library. Removing 280 duplicates left 849 unique records for title and abstract screening, of which 430 were set aside as clearly outside the scope of the review. The full texts of the remaining 419 reports were then sought; two could not be obtained, so 417 were read in full and judged against the eligibility criteria. At the full-text stage, 408 were excluded: 200 were not economic evaluations; 103 were reviews, editorials, or conference abstracts; 89 lacked extractable cost data; and 16 concerned an ineligible population. Nine studies from this stream satisfied all criteria. Screening the reference lists of relevant articles (citation searching) surfaced a further 18 reports. All were retrieved and assessed in full, and 17 were excluded and nine as non-economic evaluations, seven for absent cost data, and one for the wrong population, leaving a single eligible study. The selection process is summarized in the PRISMA 2020 flow diagram (Figure 1).

Characteristics of Included Studies

The 10 included studies were published between 2016 and 2025, originating from six countries: the United States, the Netherlands, Sweden, Norway, the United Arab Emirates and Iran (Table 2). Seven were conducted in high-income countries; the remaining three came from a single upper-middle-income country, Iran. No eligible economic evaluation was identified from a low- or lower-middle-income country, a gap that is itself a substantive finding.

**Table 2:** Characteristics of the included studies HIC: high-income country; UMIC: upper-middle-income country; KEP: kidney-exchange program; WTP: willingness to pay; DALY: disability-adjusted life years; KTx: kidney transplant; DDK: deceased donor kidney; LDT: living donor transplant; KPD: kidney paired donation; USRDS: United States Renal Data System; ESRD: end-stage renal disease; ABOi: ABO-incompatible; ABOc: ABO-compatible; PD: peritoneal dialysis; QALY: quality-adjusted life years

Ref	Author; year	Country	Income level	Study design	Sample size	Study period	Follow-up	Intervention	Comparator	Economic perspective
KPD										
16	Ahmadzada et al. (2025) [16]	USA (Mayo Clinic, MN)	HIC	Retrospective single-center cohort; standardized cost analysis (Mayo Cost Data Warehouse); Kaplan-Meier, Cox	1,266 KTx (406 DDK; 611 LDT; 151 KPD-external; 98 KPD-local)	Jan 2017- Jan 2023 (FU to 6 y)	≥1 y (survival to 6 y)	Kidney paired donation (KPD-local and KPD-external)	Deceased-donor (DDK); living-donor traditional (LDT)	Institutional/provider (standardized costs)
^17^	Glorie et al. (2022) [17]	Netherlands (Dutch KEP)	HIC	Markov model + discrete-event simulation; integer-programming allocation policies; Monte Carlo (retrospective case study)	698 enrolled pairs; 109/399 altruistic donors	2004-2016; pub. 2022	Lifetime horizon	Kidney exchange program (KEP) ± altruistic-donor enrolment; QALY-maximizing allocation	No KEP; max-transplant policy; current Dutch policy	Health value/cost-utility (discounted QALYs)
ABOi										
Ref	Author; year	Country	Income level	Study design	Sample size	Study period	Follow-up	Intervention	Comparator	Economic perspective
^18^	Axelrod et al. (2018) [18]	USA	HIC	Decision-analytic discrete event simulation (DES)	20,000 simulated ESRD patients	SRTR 2005-2015; Medicare 2003-2012	10 y (20 y secondary)	ABO-incompatible LDKT	Maintenance dialysis	Payer (Medicare)
^19^	Axelrod et al.(2016) [19]	USA	HIC	Retrospective registry cohort (USRDS-Medicare); multivariable linear regression	26,837 LDKT (271 ABOi; 62 A2i; 26,504 ABOc)	2000-Mar 2011	3 y post-transplant	ABO-incompatible LDKT	ABO-compatible LDKT	Health system/payer (Medicare)
Maintenance dialysis										
^20^	Zhang et al. (2023) [20]	Sweden	HIC	Observational cohort	693 wait-listed patients	1998-2012	3 y post-KTx	Kidney transplantation	Maintenance dialysis	Healthcare (payer)
^21^	Heldal et al. (2019) [21]	Norway	HIC	Prospective single-center cost-utility	289 candidates ≥65 y	Inclusion to 2018	1 y (long-term preliminary)	Kidney transplantation (elderly)	Waiting list	Healthcare (payer)
^22^	Soni et al. (2025) [22]	UAE (Dubai)	HIC (Gulf)	Markov cohort cost-utility; Monte Carlo PSA	Hypothetical cohort, age 45–65 y; 1,000 simulations	5-y model; publ. 2025	5 y (model)	Kidney transplantation	Hemodialysis	Third-party payer (Dubai health system); direct medical costs
^23^	Moradpour et al. (2020) [23]	Iran	UMIC	Markov cohort cost-utility (1-mo cycle); Monte Carlo PSA (1,000)	214 pts (158 HD; 56 PD) + transplant arm	Data 2017; pub. 2020	Lifetime horizon	Kidney transplantation (TX)	Peritoneal dialysis; hemodialysis	Societal (direct medical + non-medical + indirect)
^24^	Abdi et al. (2022) [24]	Iran	UMIC	Cross-sectional economic evaluation; cost–benefit analysis (contingent valuation, WTP)	266 ESRD patients	Tariffs 2021	NR	Kidney transplantation	Maintenance dialysis (comparator context)	Patient
^25^	Yaghoubifard et al. (2016) [25]	Iran	UMIC	Cross-sectional cost-effectiveness analysis; decision tree (DALY outcome)	128 patients (32 dialysis; 97 transplant)	Records 2012; pub. 2016	Onset to death (model)	Maintenance hemodialysis	Kidney transplant (cadaveric; living)	Patient and provider (hospital)

Distribution across domains was uneven: two studies addressed KPD (United States and Netherlands), two addressed ABOi living-donor transplantation (both United States), and six addressed maintenance dialysis in comparison with transplantation, spanning Sweden, Norway, the UAE, and Iran. Five were model-based: discrete-event simulations [17-18], combined with a Markov model and integer-programming allocation, Markov cohort cost-utility models [22-23], and a decision-tree cost-effectiveness analysis [25]. The remaining five were empirical analyses: retrospective registry or single-center cohorts [16,19,20], a prospective single-center cost-utility study [21], and a cross-sectional cost-benefit analysis using contingent valuation [24]. Sample sizes ranged from small single-center records (128 patients in Yaghoubifard et al. [25] to large national registries (26,837 recipients in Axelrod et al. [19], alongside hypothetical modelled cohorts (20,000 simulated patients in Axelrod et al. [18]; 1,000 simulations in Soni et al. [22]. The analytic perspective varied widely: payer or health-system [18-22], institutional/provider [16], patient [24], combined patient and provider [25], and societal [23], while Glorie et al. framed their analysis in terms of health value (discounted QALYs) rather than a costing perspective. Outcome metrics likewise differed, encompassing the ICER per QALY, cost per DALY, BCR, and willingness to pay.

Risk of Bias per Study

Critical appraisal was performed in duplicate for all included studies using design-matched instruments as specified in the Materials and methods. Overall quality ranged from moderate-low to high. The model-based evaluations with a lifetime horizon and full probabilistic analysis scored best: Glorie et al. (90%) [17], Moradpour et al. (87%) [23], and Axelrod et al. (87%) [18], the last bolstered by internal validation against observed survival rates. At the other extreme, the decision-tree analysis of Yaghoubifard et al. (60%) [25] paired a modelling structure ill-suited to a lifelong disease with small single-center data and deterministic sensitivity analysis only. The remaining studies clustered at moderate to moderate-good quality, limited most consistently by short analytic horizons, partial cost coverage and narrow analytic perspectives. The three observational cohorts additionally appraised with the NOS were at low to moderate risk of bias: 7-9 stars for Zhang et al. [20] and Axelrod et al. [19] and 6-7 for the single-center Ahmadzada et al. [16].

Economic Outcomes by Domain

KPD: Both KPD studies were conducted in high-income settings, and both concluded that paired donation is economically favorable, although neither reported a formal incremental cost-effectiveness ratio. In the Mayo Clinic cohort [16], early (30-day) costs were highest for KPD, US$124,660 for local and US$109,769 for external paired donation, compared with US$86,769 for deceased-donor and US$115,597 for traditional living-donor transplantation, driven by paired-exchange-specific expenses such as organ shipping (US$1,912-9,468), transplant-coordination services (US$5,539) and donor nephrectomy (US$2,498); however, cumulative costs converged across all groups by 365 days, and one-year graft survival was statistically comparable (KPD-external 95.2%, KPD-local 99.0%, deceased-donor 97.0%, traditional living-donor 98.6%) [16]. The authors estimated a net saving of approximately US$100,000 per paired-donation transplant relative to long-term dialysis. The Dutch kidney-exchange analysis [17] reported the program's effect in health-value terms: a kidney-exchange program increased expected discounted QALYs from 6.42 to 9.65 (an efficacy gain of 3.23 QALYs), of which the policy currently practiced and a simple transplant-maximizing policy each captured about 63% (2.11 QALYs) and a QALY-maximizing allocation 69% (2.33 QALYs) [17]. Systematically enrolling altruistic donors raised the gain further, to 4.05 QALYs (a mean of 10.27 discounted QALYs), while also reducing the disparity affecting blood-type-O recipients; living-donor transplantation was identified as the lowest-cost, dominant modality [17]. Overall, the KPD evidence indicates that paired donation and exchange achieve clinical and economic outcomes comparable to other forms of living-donor transplantation and are cost-saving relative to dialysis, but it derives entirely from two high-income health systems. The economic outcomes of each included study are summarized in Table 3.

**Table 3:** Economic evaluation USD: US Dollar; EUR: EURO; KPD: kidney paired exchange; CBA: cost-benefit analysis; QALY: quality-adjusted life years; LTx: live transplant; LDKT: living donor kidney transplant; DDKT: deceased donor kidney transplant; ABOi: ABO-incompatible; ABOc: ABO-compatible; DALY: disability-adjusted life years; ICER: incremental cost-effective ratio; WTP: willingness to pay; BCR: benefit-cost ratio; AED: Arab Emirates Dirham; KT: kidney transplant; CER: cost-effective ratio *Reported metric is BCR and net present value (NPV) from contingent valuation, not an ICER; not directly comparable to ICER/QALY values reported for other studies

Ref.	Orig. currency & year	Cost intervention	Cost comparator	Incremental cost (Δ)	Effect measure	Incremental effect	ICER/other ratio	Cost-effective?
Kidney paired donation								
16	USD (2017-2023; standardized)	KPD 0-30 d: external $109,769 ± 32,704; local $124,660 ± 88,627. 30-90 d: $5,615/$6,719. 90-366 d: $21,265/$17,259	DDK 30 d $86,769 ± 69,989; LDT $115,597 ± 28,002 (90-366 d: DDK $26,017; LDT $19,152)	30 d: KPD-local +$37,891, KPD-external +$23,000 vs DDK (P<.001); costs converge by 365 d. KPD add-ons: shipping $1,912-9,468; Tx service $5,539; nephrectomy $2,498	Graft & patient survival	1-y graft survival 95.2% (KPD-ext)/99.0% (KPD-local) vs DDK 97.0%, LDT 98.6%; 6-y patient survival 92.7%/88.8% (KPD): differences NS	NR (cost-comparison; no ICER); cites ≈$100,000 net saving/KPD Tx vs dialysis	KPD comparable cost & outcomes to traditional Tx; cost-effective vs long-term dialysis (qualitative). FLAG: single-center; standardized (not billing) costs
17	EUR (2012-2014 cited costs)	LTx lowest-cost (cited); transplant yr1 €85,127; yr2 €29,612; yr3 €20,156	Dialysis €77,566-€105,833/y (2012-2014, cited)	NR (health-value/QALY focus); interpolicy cost differences negligible (<1 Tx/y)	Discounted QALYs (lifetime)	KEP efficacy +3.23 QALY (6.42→9.65); perfect KEP +4.41; with altruists +4.05 (→10.27); QALY-max vs max-Tx +0.19 (+0.54 with altruists); reduces blood-type-O inequity	NR (health-value optimization; LTx dominant vs DTx/dialysis: cited)	Yes: KEP substantially increases health value; LTx most cost-effective; altruist enrolment adds equitable QALY gains (qualitative)
ABOi renal transplant								
18	2016 USD	USD 3,64,755 (10-y total cost, ABOi LDKT)	USD 2,92,117 (dialysis, 10-y)	+USD 72,638 vs dialysis	QALY (10-y)	+2.09 QALY (6.12 vs 4.03)	USD 59,564/QALY (ABOi cost per QALY)	Yes: cost-effective
19	2012 USD	ABOi episode USD 65,080 (ABOi+splenectomy higher)	ABOc episode USD 32,039; A2i USD 36,752	+USD 32,356 episode (adj.); pre-tx +6,584; yr1 +25,044; yr2 +10,496; yr3 +7,307	3-y patient & graft survival; Medicare payments	Patient survival 88.1% vs 93.2%; graft 76.1% vs 85.4% (ABOi lower)	NR (cost study); 10-y ABOi-ABOc increment ≈ USD 126,313	ABOi costlier than ABOc but cost-effective vs dialysis (qualitative)
Maintenance dialysis								
20	2012 EUR	€57,278 yr1 KTx; €11,513/€12,020 yr2/3	€47,775 yr1 dialysis; €47,855/€56,902 yr2/3	Yr1 +€9,502; Yr2 -€36,342; Yr3 -€44,882	Healthcare cost (3-y); QoL noted	KTx survival +~14 y (prior study); 0.024 QALY yr1	NR (cost study); KTx cost-saving from yr2	Yes: KTx cost-saving vs dialysis over 3 y
21	2018 EUR (€)	€88,100/QALY (yr1 post-Tx)	€76,495/QALY (yr1 on waiting list)	+€10,075 (Tx vs WL, yr1)	QALY (1-y)	+0.024 QALY (0.710 vs 0.686)	€419,792/QALY (1-y ICER)	No at 1 y (>threshold); favorable long-term
22	USD(~2024-25)	Transplant 5-y AED 961,323 (USD 261,941)	Hemodialysis 5-y AED 971,538 (USD 264,724)	−AED 10,215 (-USD 2,783) over 5 y (Tx saves)	QALY (5-y)	+1.3 QALY (3.4 vs 2.1)	AED 53,205 (USD 14,497)/QALY at yr 2; Tx dominant by yr 4	Yes: cost-effective from yr 2; dominant by yr 4
23	2017 USD	TX USD 16,450/y (annual avg)	PD USD 12,865/y; HD USD 13,477/y	TX vs PD +USD 3,584	QALY (lifetime; EQ-5D-5L)	TX 9.43 vs PD 6.95 vs HD 6.04; TX vs PD +2.478 QALY	TX USD 1,744/QALY;	Yes: TX cost-effective vs PD at WTP USD 12,380 (54.5% PSA)
							PD USD 1850/QALY	
							HD USD 2227/QALY	
24*	2021 USD (CBA)	KT direct cost USD 877 (avg)	Dialysis ~USD 4,176/y	NR	WTP for KT (CVM, 4 scenarios)	Mean WTP USD 4,733 (range USD 3,650-5,764)	CBA	Yes: KT net positive benefit (BCR >1 all scenarios)
							BCR 5.39; NPV USD 3,855	
25	2012 USD	Dialysis USD 15,986.9/DALY (patient persp.)	Cadaveric Tx USD 2,528.5/DALY; living Tx USD 3,181.07/DALY	Tx vs dialysis large savings	DALY (CER; dollar/DALY)	Dialysis CER 5.04 times than living-Tx, 6.15 times than cadaveric-Tx	Cadaveric Tx most cost-effective; ICER cadaveric vs living USD 5,954/DALY	Yes: transplant (cadaveric > living) far more cost-effective than dialysis; dialysis least cost-effective

ABOi transplantation: Both ABOi studies originated in the United States and adopted a payer perspective. The decision-analytic simulation [18] found ABOi living-donor transplantation to be cost-effective relative to maintenance dialysis over a ten-year horizon: although the total cost of ABOi transplantation (US$364,755) exceeded that of dialysis (US$292,117) by US$72,638, the additional 2.09 QALY gained (6.12 versus 4.03) yielded a cost per QALY of US$59,564, which is below the US$100,000/QALY willingness-to-pay threshold and lower than the US$72,476/QALY of dialysis [19]. The national registry analysis [19] quantified the incremental cost of crossing the blood-group barrier: the ABOi transplant episode cost US$65,080 versus US$32,039 for ABO-compatible (ABOc) transplantation, an adjusted increment of US$32,356, with further excess post-transplant spending in years one to three (US$25,044, US$10,496 and US$7,307) and an estimated 10-year ABOi-ABOc increment of approximately US$126,313 [20]. The two studies are concordant: ABOi transplantation is more expensive than compatible transplantation, but the increment is modest relative to, and justified by avoiding, long-term dialysis, leaving ABOi cost-effective against dialysis. As with KPD, no ABOi evidence was available from non-high-income settings.

Maintenance dialysis versus transplantation: Five of the six maintenance-dialysis studies used cost-utility or cost-effectiveness analysis based on observed costs and outcomes and were consistent in direction, finding transplantation more economical than maintenance dialysis over a multi-year or lifetime horizon. A sixth study [24] used contingent valuation to derive willingness to pay and a resulting benefit-cost ratio (BCR: 5.39), a stated-preference metric not directly comparable to the ICER/QALY-based findings of the other five, though directionally supportive of transplantation's favorable economic profile.

In Sweden [20], transplantation cost more than dialysis in the first year (€57,278 versus €47,775; +€9,502) but became markedly cheaper thereafter (-€36,342 in year two and -€44,882 in year three), a cumulative three-year saving of roughly €72,000 (about €52,000 after accounting for omitted immunosuppression and procurement costs [21]). In the UAE [22], transplantation saved AED 10,215 (US$2,783) and gained 1.3 QALYs (3.4 versus 2.1) over five years, with an incremental cost-effectiveness ratio of AED 53,205 (US$14,497)/QALY by year two and dominance by year four; probabilistic analysis favored transplantation in 100% of 1,000 simulations [23]. In Iran, the lifetime societal Markov model [23] found transplantation as the most cost-effective (US$1,744/QALY versus peritoneal dialysis), with hemodialysis as having dominated; the cross-sectional cost-benefit analysis [24] reported a mean willingness-to-pay threshold of US$4,733 for a transplant costing US$877, giving a BCR of 5.39; and the decision-tree analysis [25] found dialysis five to 14 times less cost-effective than transplantation per DALY, with cadaveric transplantation as the most cost-effective option [23-25].

Heldal et al. [21] is not directly comparable to the other five maintenance-dialysis studies: it evaluated transplantation against remaining on the waiting list (not against dialysis directly), in a cohort restricted to recipients aged 65 years or older, over a one-year horizon (€88,100/QALY versus €76,495/QALY; incremental cost-effectiveness ratio: €419,792/QALY). Given these differences in comparator, population, and horizon, this finding should not be interpreted as contradicting the transplant-favorable conclusions of the other studies but as addressing a distinct question-the short-term economic impact of transplantation timing in elderly candidates already on the pathway to transplant [21].

Findings Stratified by Resource Setting

The direction of the economic conclusion was consistent across income levels: transplantation, whether standard, paired-exchange, or ABOi, was favored over maintenance dialysis in both high- and upper-middle-income settings; the magnitude of the difference varied between settings. Absolute costs were an order of magnitude higher in high-income systems (e.g., US hemodialysis at roughly US$90,000-100,000 per year [26]) than in upper-middle-income Iran (hemodialysis approximately US$13,477 and transplantation approximately US$16,450 per year [23]). Correspondingly, incremental cost-effectiveness ratios were far lower in the Iranian studies (US$1,744/QALY in Moradpour et al. than in high-income analyses (US$14,497-US$59,564/QALY [19]), reflecting lower domestic input prices rather than any difference in the underlying economic case (Table 4).

**Table 4:** Annual direct costs of kidney replacement therapy by country and resource setting HIC: high-income country; UMIC: upper-middle-income country; LMIC: low- and middle-income country; KEP: kidney exchange program; WTP: willingness to pay; DALY: disability-adjusted life years; KTx: kidney transplant; DDK: deceased donor kidney; LDT: living donor transplant; KPD: kidney paired donation; ESRD: end-stage renal disease; ABOi: ABO-incompatible; ABOc: ABO-compatible; HD: hemodialysis; PD: peritoneal dialysis; QALY: quality-adjusted life years; USD: US Dollar; EUR: EURO; CBA: cost benefit analysis; ICER: incremental cost-effective ratio; BCR: benefit-cost ratio; AED: Arab Emirates Dirham; RMB: Renminbi; PPP: public-private partnership; CAPD: continuous ambulatory peritoneal dialysis; GDP: gross domestic product

Country		Income level		Annual HD cost (USD)		Annual PD cost (USD)		Annual post-KT maintenance cost (USD)		HD cost as % of GDP per capita		Catastrophic expenditure risk		Ref.	
High-income countries															
United States		HIC		~90,000-100,000 (Medicare prevalent-patient estimate, 2020-2024); ~40,000 if based on sessional reimbursement only		~70,000-76,000 (Medicare; PD-HD gap narrowing)		~13,700-14,000 (immunosuppression + graft maintenance; vs ~67,500 if back on dialysis)		~104-116%		Low: Medicare ESRD entitlement covers care; private-payer spending substantially higher		^[26,27,28]^	
United Kingdom		HIC		~30,800 GBP (~38,500 USD) per year		Lower than in-center HD (home-based)		~5,000 GBP (~6,300 USD) per year after first-year cost of ~17,000 GBP		~74%		Low: NHS-funded; minimal out-of-pocket		^[29]^	
Upper-middle-income countries															
China		UMIC		~11,000-12,800 (≈80,000 RMB/yr in 2010; in-center HD); older maintenance-HD estimate ~Int$35,400 (PPP)		~Int$7,800 (CAPD, PPP)		Lower than maintenance dialysis (transplant favored economically)		~85-96% (≈8× per-capita disposable income pre-2010)		Moderate: high pre-2010; improved after SHI expansion (>95% population insured)		^[30,31]^	
Thailand		UMIC		HD funded under UHC; WTP threshold ~160,000 THB (~4,600 USD)/QALY used for modality decisions		CAPD cost-effective; “PD-first” national policy		Not separately reported		Not directly reported		Low-moderate: UHC “PD-first” since 2008 limits out-of-pocket burden		^[32,33]^	
Iran		UMIC		HD-dominant system (≈97% HD); cost-utility threshold 2.5× GDP per capita (~4,100 USD, 2022)		Higher unit cost but greater effectiveness; PD expansion recommended		Not separately reported		Not directly reported		Moderate: partial public coverage		^[34]^	
Low- and middle-income countries															
India		LMIC		~3,800 per patient (public PPP, 2021-22); ~3,000 public and up to ~7,200 in private sector		~4,100 per patient (CAPD, public PPP, 2021-22)		~1,450 (≈120,000 INR/yr immunosuppression)		~110-265% (public vs private)		Very high: >80% of patients pay out-of-pocket; National Dialysis Program covers sessions only		^[35,36]^	
Pakistan		LMIC		~1,680-2,000 (2023); recurrent OOP ~1,120/yr even with state session coverage		Limited availability/data		Not separately reported		~110-135%		Very high: state covers sessions; drugs, labs, transport remain out-of-pocket		^[37]^	
Nigeria		LMIC		~8,100 (2 sessions/wk) to ~12,200 (3 sessions/wk)		Limited availability/data		Not separately reported		~500-760%		Very high: predominantly out-of-pocket; access <10-16%		^[31,38]^	
Ethiopia		LMIC		Total ~4,470/yr (2 sessions/wk; dialysis component ~2,675)		Limited availability/data		Not separately reported		~245-405%		Very high annual cost far exceeds per capita		[39]	

Annual hemodialysis cost as a proportion of GDP per capita rises steeply with declining national income, from 74-116% in the UK and US to 85-96% in China, to 110-265% in India, to 500-760% in Nigeria (Table 4). Out-of-pocket exposure shifts from minimal (under Medicare-type or universal coverage) to catastrophic, with more than 80% of dialysis patients paying out of pocket in low- and lower-middle-income settings. The economic and equity case for transplantation, and for KPD as the most efficient route to it, therefore strengthens as resources diminish. Yet no included evaluation originated from a low- or lower-middle-income country, which is itself a principal finding of this review.

Reporting Biases and Certainty of Evidence

**Figure 2:**
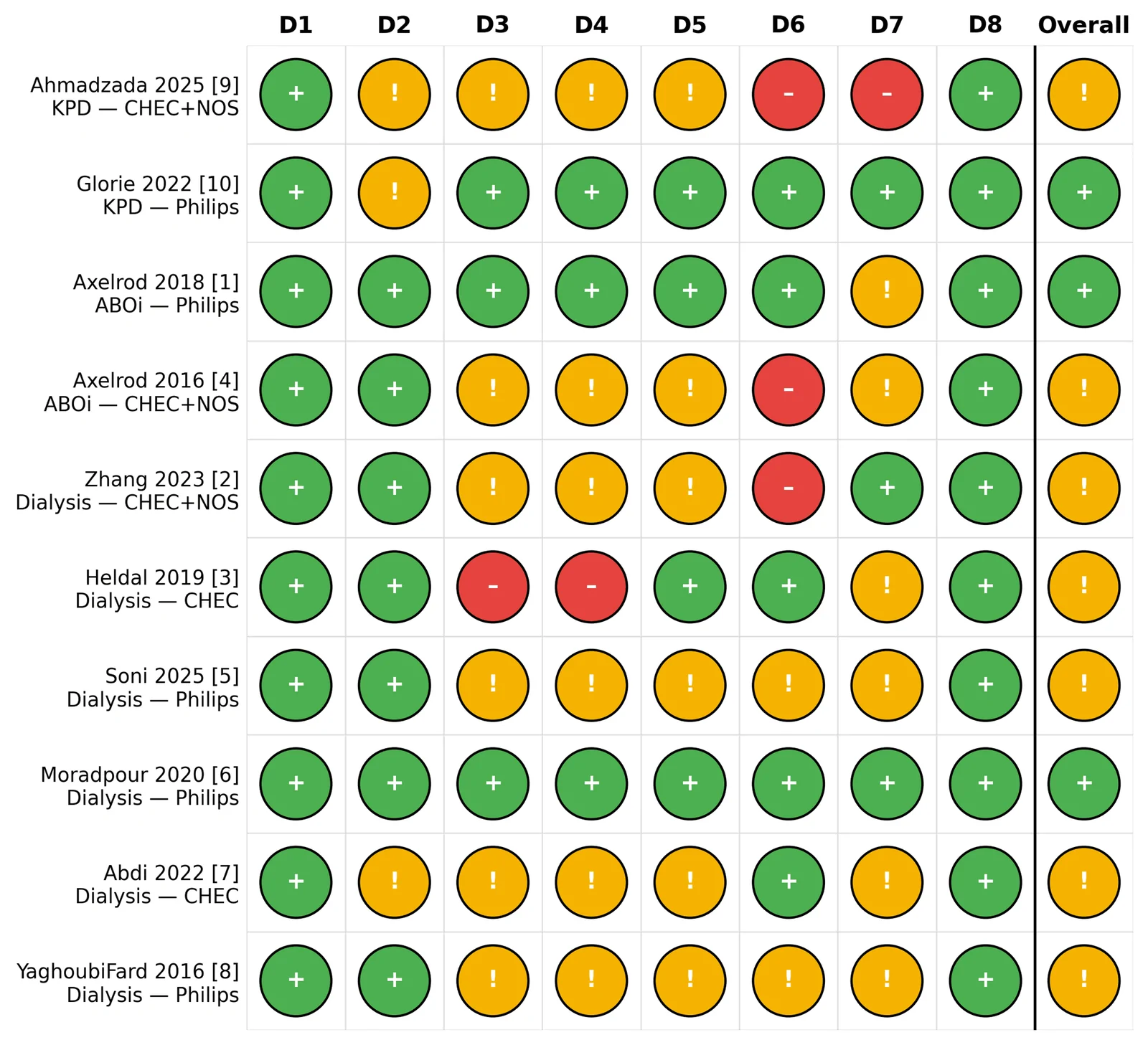
Risk of bias assessment of included studies D1: population and comparators; D2: perspective; D3: time horizon; D4: cost measurement; D5: outcome measurement; D6: analytic approach; D7: uncertainty analysis; D8: reporting and conflict of interest Each dot represents a domain-level judgement: green = low risk of bias; amber = some concerns; red = high risk of bias

Formal statistical assessment of reporting bias, such as funnel-plot asymmetry or Egger’s test, was neither feasible nor appropriate, given the small number of studies within each domain, the diversity of outcome metrics and the narrative (non-meta-analytic) synthesis. Reporting and publication bias were therefore considered qualitatively. The possibility that economic analyses with unfavorable or null findings are less likely to be published cannot be excluded; were such bias present, it would tend to reinforce rather than reverse the consistently transplantation-favoring direction observed here, and the very small KPD and ABOi evidence bases (two studies each) are the most vulnerable to it. Figure 2 represents risk of bias across all included studies, grouped by review domain (KPD, ABOi transplantation, maintenance dialysis). Domain-level judgements were derived by mapping items from the CHEC-list and Philips checklist; the design matched the instruments used for trial-/cohort-based and model-based economic evaluations, respectively, onto these eight common risk-of-bias domains; each scored using the original item-level appraisal grade (Y = 1; p = 0.5; N/U = 0) and classified as low risk (≥75%), some concerns (55-74%), or high risk (<55%).

Certainty was characterized narratively, as GRADE is not designed for economic evaluations. Overall confidence was judged low to moderate. It was highest for the lifetime-horizon, fully probabilistic models [17,18,23] and lowest for the decision-tree analysis of Yaghoubifard et al. [25]. Three recurring limitations reduced confidence across the remaining studies. First, short analytic horizons (one year in Heldal et al. [21] and three to five years in Zhang et al. [20], Axelrod et al. [19], and Soni et al. [22]) systematically underestimate the long-term value of transplantation and directly explain the single unfavorable ICER in this review. Second, the preponderance of partial evaluations reports costs and survival separately, without an integrated cost-utility estimate [16,19,20]. Third, marked heterogeneity in analytic perspective and cost coverage, with immunosuppression, organ-acquisition, or indirect costs, was excluded from several analyses. Generalizability was further constrained by reliance on single-center, single-payer, or single-registry data. None of these limitations undermines the directional finding. The cost-effectiveness conclusion in favor of transplantation was consistent across study designs, analytic perspectives, and income settings, a robustness that increases confidence in the qualitative conclusion even where point-estimate certainty is limited.

Discussion

Principal Findings

Across every analysis in this review, transplantation was economically preferable to maintenance dialysis, regardless of income level, analytic design, currency, or outcome metric (see Results for full figures). This directional consistency held despite absolute costs and ICERs differing by an order of magnitude between high-income and upper-middle-income settings. What it did not hold across was geography: the evidence base was concentrated in high-income systems, thin in the KPD and ABOi domains, and absent from low- and lower-middle-income countries.

KPD: The economic rationale for KPD is essentially a counterfactual argument: it converts an otherwise nontransplantable incompatible pair into a compatible living-donor transplant, displacing long-term dialysis, desensitization, or both. The two included studies address this value at different levels: Ahmadzada et al. at the patient level, establishing no durable cost penalty against other transplant modalities, and Glorie et al. at the system level, establishing that allocation policy and altruistic-donor enrolment maximize population health value and equity together. Both support KPD as a first-line strategy rather than a fallback, though this evidence is methodologically partial (neither study reports a formal ICER) and derives entirely from two high-income systems. Kidney-exchange programs have expanded substantially in middle-income settings, including some of the largest single-center programs in India, where deceased-donor pools are limited, and the out-of-pocket burden of both dialysis and desensitization is high [40], yet no accompanying cost-utility analysis exists; this is the most important evidence gap identified by this review and suggests the barrier to economic evidence generation in LMICs is institutional and data-related, not a reflection of KPD's relevance.

ABOi transplantation and the case for paired donation: The ABOi findings complement the KPD argument: transplantation is cost-effective compared with dialysis but markedly more expensive than compatible transplantation, with modestly lower survival, a pattern corroborated at the meta-analytic scale [41,42]. This cost penalty is a feature of the desensitization protocol itself, not of the recipient population or health system, and there is little reason to expect it to disappear in non-high-income settings. Because KPD achieves a compatible graft without this penalty, the two strategies are better viewed as sequential rather than competing: KPD attempted first, and ABOi or HLA-incompatible desensitization reserved for pairs that cannot be matched within an exchange, a sequencing reinforced by the enrichment of exchange pools with blood-type-O recipients, which altruistic-donor chains are uniquely positioned to relieve.

Maintenance dialysis and the resource-setting gradient: The maintenance dialysis studies provide the foundation for the transplantation case and were consistent in direction, with the lone exception of Heldal et al. [21], which is attributable to its distinct comparator (waiting list, not dialysis), population (elderly, ≥65y), and one-year horizon rather than a contrary economic signal. Lower incremental cost-effectiveness ratios in Iran reflect lower domestic input prices, not a weaker economic argument, and should be read against context-specific thresholds rather than a single high-income benchmark [43]. This gradient is visible even outside the 10 included studies: multinational data place the global median in-center hemodialysis cost at roughly US$19,380, with catastrophic household expenditure repeatedly documented among uninsured dialysis-dependent households in South Asia [44,45]. None of this contradicts this review's findings; every mechanism that makes dialysis expensive in a low-resource setting strengthens, rather than weakens, the case for transplantation and specifically KPD as the least resource-intensive route to it.

Strengths and Limitations

This review has one principal strength: the integration of three transplantation-economics domains that are seldom synthesized together, appraised with design-matched, dual-reviewer instruments (CHEC, the Philips checklist, the NOS) and read through an explicit equity and resource-setting lens.

The limitations are no less clear, and they temper the conclusions accordingly. The evidence base is small; 10 studies and two studies each in the KPD and ABOi domains, the ABOi evidence confined to a single country, and the entire non-high-income contribution coming from one upper-middle-income setting. No eligible study came from a low- or lower-middle-income country at all. Heterogeneity in design, method, and economics precluded meta-analysis. Several studies were partial evaluations with short horizons or narrow perspectives, and the KPD evidence in particular reports no formal ICER and is wholly high-income. The outcomes are ICER/QALY, cost/DALY, BCR, willingness to pay, net present value, and pure cost comparison. These are not convertible to one another. So, SWiM reporting was done. 

This review was not registered with PROSPERO prior to conduct, which limits transparency regarding prespecification of our methods and outcomes and precludes independent verification that our review question, eligibility criteria, and analysis plan were not modified after data collection began. We note this as a limitation of our review process.

Implications for Practice and Policy

Transplantation should be prioritized as the economically dominant form of kidney replacement therapy wherever it is clinically feasible. KPD should be expanded and placed ahead of desensitization-based ABOi or HLA-incompatible transplantation for any incompatible pair that can be matched within an exchange. Exchange registries should allocate to maximize health value, not transplant count, and should actively recruit altruistic and nondirected donors, as the Dutch data show this improves efficiency and equity together, at negligible cost, and disproportionately benefits blood-type-O recipients. The argument is strongest where resources are tightest: in lower-resource systems, the steep affordability gradient and the heavy out-of-pocket burden of dialysis make investment in living-donor and paired-donation infrastructure, paired with financial-protection mechanisms, among the most compelling uses of constrained health spending available.

Implications for Research

The single most pressing research need is economic evaluation of KPD and ABOi transplantation in upper-middle-, lower-middle-, and low-income settings, where these strategies are increasingly practiced yet economically unstudied. Future KPD work should abandon partial cost descriptions for full cost-utility analyses with lifetime horizons and reported ICERs and should model paired donation directly against desensitization to put a number on the value of avoiding antibody reduction. Standardized reporting of analytic perspective and public-private partnership (PPP)-adjusted costs would make cross-country comparison possible. Equity-oriented analyses of blood-type-O disadvantage, altruistic-donor chains, and out-of-pocket exposure would align the economic evidence with the access and fairness questions that dominate kidney replacement therapy wherever resources are scarce.

## Conclusions

This review confirms that kidney transplantation, whether achieved through paired exchange or ABOi living donation, remains economically superior to maintenance dialysis across every resource setting examined. KPD emerged as the most favorable strategy, combining direct cost savings, meaningful quality-of-life gains, and avoidance of desensitization-related expenditure. ABOi transplantation, while consistently more expensive than compatible transplantation, still met conventional cost-effectiveness thresholds relative to dialysis. Notably, the magnitude of cost-effectiveness ratios varied by setting, substantially lower in Iran than in high-income countries, but this reflects differences in absolute healthcare pricing rather than a weaker underlying economic case for transplantation. The near-complete absence of data from low- and lower-middle-income countries represents a critical evidence gap and is particularly concerning given that affordability constraints, and therefore the practical relevance of this economic argument, are likely most acute in precisely those settings.

## Appendices

Comprehensive literature search strategy

Comprehensive search strategy for Embase available in Table 5.

**Table 5:** Search strategy for Embase

Database	Search string
Embase	1. exp kidney transplantation/ or kidney transplant*.ti,ab,kw. or renal transplant*.ti,ab,kw.
	2. kidney paired donation.ti,ab,kw. or paired kidney exchange.ti,ab,kw. or kidney paired exchange.ti,ab,kw. or paired donation.ti,ab,kw. or KPD.ti,ab,kw. or kidney exchange programme.ti,ab,kw.
	3. ABO incompatible.ti,ab,kw. or ABO-incompatible.ti,ab,kw. or ABOi.ti,ab,kw. or blood group incompatible.ti,ab,kw. or blood type incompatible.ti,ab,kw.
	4. exp maintenance hemodialysis/ or exp peritoneal dialysis/ or maintenance dialysis.ti,ab,kw. or haemodialysis.ti,ab,kw. or hemodialysis.ti,ab,kw. or peritoneal dialysis.ti,ab,kw.
	5. 2 or 3 or 4
	6. 1 and 5
	7. exp cost/ or exp cost effectiveness analysis/ or exp cost benefit analysis/ or exp cost utility analysis/ or cost.ti,ab,kw. or cost-effectiveness.ti,ab,kw. or economic evaluation.ti,ab,kw. or cost-benefit.ti,ab,kw. or cost-utility.ti,ab,kw. or health economics.ti,ab,kw. or ICER.ti,ab,kw. or QALY.ti,ab,kw. or "quality adjusted life year".ti,ab,kw. or DALY.ti,ab,kw. or "disability adjusted life year".ti,ab,kw. or willingness to pay.ti,ab,kw. or WTP.ti,ab,kw. or budget impact.ti,ab,kw. or financial burden.ti,ab,kw. or resource utilisation.ti,ab,kw. or resource utilization.ti,ab,kw. or incremental cost.ti,ab,kw.
	8. 6 and 7

Comprehensive search strategy for PubMed/MEDLINE is available in Table 6.

**Table 6:** Search strategy for PubMed/MEDLINE MeSH: Medical Subject Headings; KPD: kidney paired donation; ABOi: ABO-incompatible; ABOc: ABO-compatible; LMIC: low- and middle-income country; WTP: willingness to pay; DALY: disability-adjusted life years; QALY: quality-adjusted life years; ICER: incremental cost-effective ratio

Database	Search string
PubMed (MEDLINE)	("kidney transplantation"[MeSH Terms] OR "renal transplantation"[tiab] OR
	"kidney transplant"[tiab])
	AND
	("kidney paired donation"[tiab] OR "paired kidney exchange"[tiab] OR
	"kidney paired exchange"[tiab] OR "paired donation"[tiab] OR
	"kidney exchange programme"[tiab] OR "KPD"[tiab]
	OR
	"ABO incompatible"[tiab] OR "ABO-incompatible"[tiab] OR "ABOi"[tiab] OR
	"blood group incompatible"[tiab] OR "blood type incompatible"[tiab]
	OR
	"maintenance dialysis"[tiab] OR "haemodialysis"[tiab] OR "hemodialysis"[tiab] OR
	"peritoneal dialysis"[tiab] OR "dialysis"[MeSH Terms])
	AND
	("cost"[tiab] OR "costs and cost analysis"[MeSH Terms] OR
	"cost-effectiveness"[tiab] OR "cost effectiveness"[tiab] OR
	"cost-benefit"[tiab] OR "economic evaluation"[tiab] OR
	"cost-utility"[tiab] OR "cost utility"[tiab] OR
	"health economics"[tiab] OR "economic analysis"[tiab] OR
	"incremental cost"[tiab] OR "ICER"[tiab] OR
	"quality-adjusted life year"[tiab] OR "QALY"[tiab] OR
	"disability-adjusted life year"[tiab] OR "DALY"[tiab] OR
	"willingness to pay"[tiab] OR "WTP"[tiab] OR
	"budget impact"[tiab] OR "financial burden"[tiab] OR
	"resource utilisation"[tiab] OR "resource utilization"[tiab])
	AND
	("low-income"[tiab] OR "middle-income"[tiab] OR "developing countr*"[tiab] OR
	"resource-limited"[tiab] OR "resource-constrained"[tiab] OR
	"low and middle income"[tiab] OR "LMIC"[tiab] OR
	"resource setting*"[tiab] OR "healthcare setting*"[tiab] OR
	"economic setting*"[tiab])
	Filters: English language; humans

Critical appraisal of included studies

All 10 included studies were appraised in duplicate from the full-text reports using design-matched instruments:

The Consensus Health Economic Criteria (CHEC) list for the trial- and cohort-based economic evaluations; the Philips checklist for the model-based (decision-analytic) evaluations; and the Newcastle-Ottawa Scale (NOS) for those studies whose primary design was a comparative observational cohort. Studies were not excluded on the basis of appraisal; the results are used to weigh the interpretation of the synthesis. Scoring legends for critical appraisal are shown in Table 7.

**Table 7:** Scoring legend QALY: quality-adjusted life years; ICER: incremental cost-effective ratio

Symbol	Meaning	Interpretation in this appraisal
Y	Yes, criterion met	The criterion is fully satisfied as reported in the study (e.g., perspective clearly stated and appropriate; an incremental analysis with an ICER performed)
P	Partially met	The criterion is addressed but incompletely, or only under limiting assumptions, for example, relevant costs identified but some components excluded, or sensitivity analysis limited to deterministic (one-way) tests with no probabilistic analysis
N	No, not met	The criterion is not satisfied as reported, for example, outcomes not valued in utility terms (no QALY), or a time horizon too short to capture the relevant costs and effects
U	Unclear/not reported	The full text does not provide enough information to make a judgment (e.g., model internal validation neither described nor referenced). Not a negative judgment in itself, but a reporting gap

The summary of overall methodological quality of all included studies is shown in Table 8.

**Table 8:** Methodological quality CHEC: The Consensus Health Economic Criteria; NOS: Newcastle-Ottawa Scale; KPD: kidney paired exchange; PSA: probabilistic sensitivity analysis; CUA: cost-utility analysis; QALY: quality-adjusted life years; DALY: disability-adjusted life years; ICER: incremental cost-effective ratio; WTP: willingness to pay; ABOi: ABO-incompatible; ABOc: ABO-compatible; PSM: propensity score matching; IPWRA: inverse probability weighting with regression adjustment; SRTR: Scientific Registry of Transplant Recipients

Study (ref)	Domain	Design	Instrument	Summary score	Overall rating	Principal strengths/concerns
Ahmadzada et al., 2025 [16]	KPD	Retrospective single-center cohort; standardized cost analysis	CHEC + NOS	58% (11/19) · NOS 6-7/9	Moderate	Large, rigorous clinical cohort; but partial evaluation (cost description + separate survival, no ICER), provider-only standardized (non-billing) costs, ~1-y cost horizon, single-center
Glorie et al., 2022 [17]	KPD	Markov + discrete-event simulation; integer-programming allocation	Philips	90% (13.5/15)	Good/high	Sophisticated lifetime model, primary Dutch data, extensive scenario/uncertainty analysis and blood-type/age equity subgroups; but a health-value (QALY) optimization rather than a full cost-utility (costs contextual), single-country
Axelrod et al., 2018 [18]	ABOi	Decision-analytic discrete-event simulation	Philips	87% (13/15)	Good	Robust SRTR/Medicare data, internal validation against observed rates, 3% discounting, 10/20-y horizon; only US-payer specific
Axelrod et al., 2016 [19]	ABOi	Retrospective registry cohort; multivariable OLS	CHEC + NOS	66% (12.5/19) · NOS 7-8/9	Moderate-good	Large national registry, actual Medicare payments, careful adjustment; partial evaluation (no cost-utility), 3-y horizon with 10-y extrapolation, excludes acquisition/indirect costs
Zhang et al., 2023 [20]	Dialysis	Observational cohort; PSM + IPWRA	CHEC + NOS	68% (13/19) · NOS 8-9/9	Moderate-good	Methodologically strong cost analysis (propensity methods, bootstrap CIs), payer perspective; partial evaluation (no ICER/primary QALY), 3-y horizon, excludes drug and procurement costs.
Heldal et al., 2019 [21]	Dialysis	Prospective single-center cost-utility	CHEC	74% (14/19)	Moderate-good	Genuine patient-level CUA (SF-6D, before-after), transparent; key weaknesses: 1-y horizon (drives the unfavourable ICER), costs borrowed from Sweden (not measured), no PSA, single-center elderly subgroup
Soni et al., 2025 [22]	Dialysis	Markov cohort cost-utility; Monte Carlo PSA	Philips	67% (10/15)	Moderate-good	Proper CUA with PSA and local Dubai costs; weaker on short 5-y horizon, hypothetical cohort, utilities/transition probabilities from international sources, simple 3-state model, explicitly nongeneralizable
Moradpour et al., 2020 [23]	Dialysis	Markov cohort cost-utility (1-mo cycle); Monte Carlo PSA	Philips	87% (13/15)	Good	Lifetime societal Markov, primary local cost + EQ-5D-5L data, full PSA/CEAC/tornado, multi-country comparison; weaker on small sample (n = 214), no internal validation, possibly borrowed utility value set
Abdi et al., 2022 [24]	Dialysis	Cross-sectional cost-benefit (contingent valuation/WTP)	CHEC	71% (13.5/19)	Moderate	Reasonable CBA with validated CVM instrument and regression, strong equity discussion; inherent CVM hypothetical bias, patient-only perspective, direct costs only, cross-sectional (no true time horizon)
Yaghoubifard et al., 2016 [25]	Dialysis	Decision-tree cost-effectiveness (DALY outcome)	Philips	60% (9/15)	Moderate-low	Two perspectives and external comparison; but decision-tree structure is suboptimal for a chronic lifelong disease, small single-center 2012 data, deterministic sensitivity only (no PSA), some internal reporting inconsistencies

The search string for the Cochrane Library is shown in Table 9.

**Table 9:** Search string for Cochrane Library QALY: quality-adjusted life years; DALY: disability-adjusted life years; ICER: incremental cost-effective ratio; WTP: willingness to pay; ABOi: ABO-incompatible; ABOc: ABO-compatible

Database	Search string
Cochrane Library (CENTRAL)	#1 "kidney transplant*":ti,ab,kw OR "renal transplant*":ti,ab,kw
	#2 "kidney paired donation":ti,ab,kw OR "paired kidney exchange":ti,ab,kw OR "kidney paired exchange":ti,ab,kw OR "paired donation":ti,ab,kw OR "KPD":ti,ab,kw OR "kidney exchange programme":ti,ab,kw
	#3 "ABO incompatible":ti,ab,kw OR "ABO-incompatible":ti,ab,kw OR "ABOi":ti,ab,kw OR "blood group incompatible":ti,ab,kw OR "blood type incompatible":ti,ab,kw
	#4 "maintenance dialysis":ti,ab,kw OR "haemodialysis":ti,ab,kw OR "hemodialysis":ti,ab,kw OR "peritoneal dialysis":ti,ab,kw
	#5 #2 OR #3 OR #4
	#6 #1 AND #5
	#7 "cost":ti,ab,kw OR "cost-effectiveness":ti,ab,kw OR "economic evaluation":ti,ab,kw OR "cost-benefit":ti,ab,kw OR "cost-utility":ti,ab,kw OR "health economics":ti,ab,kw OR "ICER":ti,ab,kw OR "QALY":ti,ab,kw OR "quality-adjusted life year":ti,ab,kw OR "DALY":ti,ab,kw OR "disability-adjusted life year":ti,ab,kw OR "willingness to pay":ti,ab,kw OR "WTP":ti,ab,kw OR "budget impact":ti,ab,kw OR "financial burden":ti,ab,kw OR "resource utilisation":ti,ab,kw OR "resource utilization":ti,ab,kw OR "incremental cost":ti,ab,kw
	#8 #6 AND #7
	Limits: English language; Trials, Reviews, and Economic Evaluations (CENTRAL); Publication years 2016–2025
